# Does internal limiting membrane peeling during epiretinal membrane surgery induce microscotomas on microperimetry? Study protocol for PEELING, a randomized controlled clinical trial

**DOI:** 10.1186/s13063-020-04433-9

**Published:** 2020-06-08

**Authors:** Jean-Baptiste Ducloyer, Juliette Ivan, Alexandra Poinas, Olivier Lebreton, Alexandre Bonissent, Paul Fossum, Christelle Volteau, Ramin Tadayoni, Catherine Creuzot-Garchet, Yannick Le Mer, Julien Perol, June Fortin, Anne Chiffoleau, Fanny Billaud, Catherine Ivan, Michel Weber

**Affiliations:** 1 Department of Ophthalmology, CHU Nantes, Nantes, France; 2 Clinical Investigation Centre CIC1413, INSERM and CHU Nantes, Nantes, France; 3 Sponsor Department, CHU Nantes, Nantes, France; 4 Ophthalmology Department, Hôpital Lariboisière, AP-HP, Université Paris 7 - Sorbonne Paris Cité, Paris, France; 5 Ophthalmology Department, CHU Dijon, Dijon, France; 6 Fondation Ophtalmologique Adolphe de Rothschild, Paris, France; 7 Ophthalmology Department, Polyclinique de l’Atlantique, Saint-Herblain, France

**Keywords:** Idiopathic epiretinal membrane, Internal limiting membrane, Peeling, Microscotomas

## Abstract

**Background:**

The epiretinal membrane (ERM) is a degenerative condition associated with age, which can cause loss of vision and/or metamorphopsia. The treatment of symptomatic ERM involves surgical removal including a vitrectomy followed by peeling of the ERM using a microforceps. As the internal limiting membrane (ILM) is adherent to the ERM, it is sometimes removed with it (spontaneous peeling). If ILM remains in place, it can be removed to reduce ERM recurrence. However, it is important to clarify the safety of ILM peeling, while it increases surgical risks and cause histological disorganization of the retina that can lead to microscotomas, may be responsible for definitive visual discomfort.

**Methods:**

PEELING is a prospective, randomized, controlled, single-blind, and multicentered trial with two parallel arms. This study investigates the benefit/risk ratio of active ILM peeling among individuals undergoing ERM surgery without spontaneous ILM peeling. Randomization is done in the operating room after ERM removal if ILM remains in place. After randomization, the two groups—“active peeling of the ILM” and “no peeling of the ILM”—are compared during a total of three follow-up visits scheduled at month 1, month 6, and month 12. Primary endpoint is the difference in microscotomas before surgery and 6 months after surgery. Patients with spontaneous peeling are not randomized and are included in the ancillary study with the same follow-up visits and the same examinations as the principal study.

Relevant inclusion criteria involve individuals aged > 18 years living with idiopathic symptomatic ERM, including pseudophakic patients with transparent posterior capsule or open capsule or lensed patients with age-related cataracts. The calculated sample size corresponds to 53 randomized eyes (one eye/patient) per arm that means 106 randomized eyes (106 randomized patients) in total and a maximum of 222 included patients (116 spontaneous peeling).

**Discussion:**

ILM peeling is often practiced in ERM surgery to reduce ERM recurrence. It does not impair postoperative visual acuity, but it increases the surgical risks and causes anatomical damages. If active ILM peeling is significantly associated with more microscotomas, it may contraindicate the ILM peeling during primitive idiopathic ERM surgery.

**Trial registration:**

ClinicalTrials.gov, NCT02146144. Registered on 22 May 2014. Recruitment is still ongoing.

## Administrative information

Note: the numbers in curly brackets in this protocol refer to SPIRIT checklist item numbers. The order of the items has been modified to group similar items (see http://www.equator-network.org/reporting-guidelines/spirit-2013-statement-defining-standard-protocol-items-for-clinical-trials/).

**Table:** 

**Title {1}**	Prospective, randomized, controlled and single-blind study assessing the benefit/risk ratio of internal limiting membrane (ILM) peeling during Epiretinal Membrane (ERM) surgery.
**Trial registration {2a and 2b}**	Registration number NCT02146144, first published on 22 May, 2014. https://clinicaltrials.gov/ct2/show/NCT02146144
**Protocol version {3}**	The updated protocol is at version 7 on 06 February 2020.
**Funding {4}**	This study is supported by a grant from the French Ministry of Health awarded in **2013** (under the Hospital Clinical Research Program), no. **13-0170**.
**Author detail {5a}**	Jean-Baptiste Ducloyer, Olivier Lebreton, Alexandre Bonissent, Paul Fossum, Fanny Billaud, Catherine Ivan, Michel Weber belong to the CHU Nantes, Ophthalmology Department and the Clinical Investigation Centre CIC1413 (INSERM and CHU Nantes). Juliette Ivan and Alexandra Poinas belong exclusively to the Clinical Investigation Centre CIC1413 (INSERM and CHU Nantes). Christelle Volteau, June Fortin and Anne Chiffoleau belong to the CHU Nantes, Sponsor Department. Ramin Tadayoni, Catherine Creuzot-Garchet, Yannick Le Mer, Julien Perol, are principal investigators, belong respectively to the Ophthalmology Department of Lariboisière Hospital (AP-HP), CHU de Dijon, the Rothschild Foundation and the Polyclinique de l’Atlantique.
**Name and contact information for the trial Sponsor {5b}**	June Fortin is the sponsor project manager and she’s coordinating the logistics of the trial. As already mentioned, she belongs to the Sponsor Department of CHU de Nantes. Any request for Peeling information can be made via this e-mail address: BP-direction-de-la-recherche@chu-nantes.fr .
**Role of sponsor {5c}**	All the submissions/declarations were made by the Sponsor Department at CHU Nantes, which of course manages the quality of the data collected. The data collected during the study will be processed electronically in accordance with the requirements of the CNIL, the French Data Protection Authority and with the European and French regulations regarding the safety concerns. Requests for substantial modifications of the protocol should be addressed by the sponsor for approval or notification to French regulatory authorities and/or the Ethical Review Board concerned in compliance with Law 2004–806 of 9 August, 2004 and its implementing decrees.

## Introduction

### Background and rationale {6a}

The epiretinal membrane (ERM) is a degenerative condition associated with age, characterized by a fibrocellular proliferation developing at the surface of the macula. The treatment of symptomatic ERM remains surgical removal including a vitrectomy followed by peeling of the ERM using a microforceps. As the internal limiting membrane (ILM) is adherent to the ERM, it is sometimes removed with it (spontaneous peeling). If it remains in place, the relevance of ILM intentional removal (“active peeling”) remains controversial.

The surgeons often peel the ILM as an adjuvant action expected to increase the success rate of the surgery. Some observational studies found that the risk of recurrence is reduced from 7%–23% without ILM peeling to 0%–4% with ILM peeling during idiopathic ERM removal [1–6]. In a meta-analysis, Azuma et al. [7] found a significant reduction of recurrence rate in case of ILM peeling during idiopathic ERM surgery (odds ratio 0.25; 95% confidence interval 0.12–0.49). Two randomized clinical trials compared ILM peeling or not: De Novelli et al. found a similar difference after 6 months (4% of recurrence with ILM peeling and 17% without) but did not reach significance; Tranos et al. did not found any recurrence in both groups after 12 months [8]. In case of recurrent ERM, vision is impaired in only half of the patients; if visual loss does occur, a second ERM surgery is possible [9].

The peeling of the ILM does not alter nor improve postoperative visual acuity (VA) [7–11]. This additional procedure lengthens the operation, increases the surgical risks (phototraumatism, retinal tear, central or eccentric macular hole) [12–14] and the risk of histological disorganization of the retina [15], which can result in one or more microscotomas with possible definitive visual discomfort for the patients. ILM is formed by Muller cells end-feet and ILM peeling results in significant damage of Muller cells [16]. Swelling of the arcuate fiber layer (SANFL) [17] and dissociated optic nerve fiber layer (DONFL) [18, 19] are well-described retinal changes due to ILM peeling but no impairment of VA has been linked to these findings.

Thanks to microperimetry, it is now possible to study more precisely the functional impairment in various retinal pathologies [20], especially with optical coherence tomography (OCT) scanning laser ophthalmoscope (SLO) microperimetry which couples microperimetry data with OCT data [21]. A retrospective study using microperimetry by Tadayoni et al. found that ILM peeling may reduce retinal sensitivity and significantly increase the incidence of microscotomas after macular surgery [22]. These abnormalities may explain the visual discomfort reported by some patients undergoing ERM and are not detected by measurement of VA and/or visual field. In a retrospective study, Deltour et al. found that active peeling was associated with more numerous and deeper microscotomas than spontaneous peeling [23]. Their localization seemed to fit the gripping areas of the ERM and ILM.

The purpose of this study is to clarify the benefit/risks ratio of the ILM peeling during ERM surgery. Microperimetry takes a central role to search for the number and type of microscotomas induced by the surgery. This study also further refines the analysis of anatomical abnormalities visualized by spectral domain optical coherence tomography (SD-OCT) and their correlation with the visual outcome of this surgery and the presence of microscotomas.

If active ILM peeling is significantly associated with more microscotomas, it may contraindicate the ILM peeling during primitive idiopathic ERM surgery.

### Objectives {7}

The main objective is to compare differences in microscotomas between the “active peeling of the ILM” group and the “no ILM peeling” group between the inclusion visit and 6 months after surgery. The main criterion is the difference between the number of microscotomas (sensitivity < 10 dB).

The secondary objectives are the difference of anatomical and functional changes of the retina between the two groups at 1, 6, and 12 months and the rate of recurrence of ERM at 12 months. Various endpoints are assessed: VA measurements (ETDRS scale) and near vision (Parinaud); microperimetry; questionnaires of visual discomfort; and SD-OCT analysis.

An ancillary study is also conducted and concerns the patients who undergo spontaneous peeling of the ILM during the surgery, as they cannot be included in the main study. The aim is to compare primary and secondary endpoints between patients with spontaneous peeling and patients in the active peeling group at inclusion visit and 1, 6, and 12 months after surgery.

### Trial design {8}

PEELING is a two-arm prospective, multicentered, controlled, randomized, and single-blind trial associated with an ancillary study about patients with spontaneous ILM peeling.

## Methods: participants, interventions, and outcomes

### Study setting {9}

This study is multicentered and national; patients are recruited in the six national ophthalmology services of the Nantes University Hospital, of the Lariboisière Hospital, of the Dijon University Hospital, of the Fondation Ophtalmologique Adolphe de Rothschild, of the private hospital of Saint-Herblain (Polyclinique de l’Atlantique), and of the Sourdille private hospital of Nantes.

As stated before, the recruitment is scheduled on six hospitals centers.

### Eligibility criteria {10}

The patients experiencing ERM are usually aged > 60 years. The etiology is mostly idiopathic but can be secondary to various diseases (diabetic retinopathy, inflammation [uveitis], trauma, recent eye surgery, retinal detachment or tear). Idiopathic ERM affect about 7% of patients after the age of 50 years according to the Blue Mountains Eye Study (conducted on an Australian population) [24]. They are often asymptomatic and not treated. In this study, 28% of patients had a loss of VA and 7.1% had metamorphopsia (distortion of lines) [24]. They then needed surgery.

The study population concerns only patients with symptomatic idiopathic ERM, responsible for symptoms such as decreased VA and metamorphopsia.

Other inclusion criteria are patients aged ≥ 18 years and women without childbearing potential or with active contraception (intrauterine device, contraceptive pill, or contraceptive implant). For patients with both eyes affected, the treated eye in the protocol is the one that is the most severely affected.

For the patients with preoperative lens opalescence, a cataract surgery is performed. As ERM mainly affects patients aged > 60 years, most of them already have preoperative bilateral lens opalescence. The worsening of the cataract in the year after vitrectomy is the most common surgery complication of ERM. The onset or continued worsening of postoperative cataracts may alter visual recovery and disrupt the assessment of visual function including retinal sensitivity microperimetry [25–27]. Furthermore, combined cataract surgery and vitrectomy for ERM is a commonly used technique that provides good functional results [28].

The main non-inclusion criteria are the presence of another pathology: age-related macular degeneration; retinal vein occlusion; diabetic retinopathy; glaucoma with macular visual field defect; or uveitis. Patients who underwent any recent eye injuries or eye surgeries (> 6 months) are also excluded. All the inclusion and non-inclusion criteria are in the Table 1.

**Table 1:** Inclusion and non-inclusion criteria

Inclusion criteria	Non-inclusion criteria
✓ Adult patients (aged > 18 years), woman without childbearing potential or active contraception (intrauterine device, contraceptive pill, or contraceptive implant)	✓ Patient with other retinal pathologies such as age-related macular degeneration (“AMD”), retinal vein occlusion, diabetic retinopathy, glaucoma with macular visual field defect
✓ Patients with an idiopathic symptomatic epimacular membrane; for patients with both eyes affected, the treated eye in the protocol will be the one that is most severely affected	✓ Patients with uveitis or a history of uveitis
✓ Pseudophakic patients with transparent posterior capsule or open capsule or lensed patients with age-related cataracts	✓ Patients with any recent eye injuries or eye surgeries (< 6 months)
✓ Patients with social security	✓ Patients participating in interventional clinical trial
✓ Patients able to understand and follow the trial instructions	✓ Pregnant or breastfeeding women
✓ Patients who have signed an informed consent	✓ Vulnerable people: persons deprived of liberty, under trusteeship, or under curatorship

### Who will take informed consent {26a}

Patient’s written consent was obtained by the investigator before any study-specific procedures. Participation is voluntary, individuals may withdraw at any stage, and participation does not affect the treatment of the individual.

### Additional consent provisions for collections and use of participant data and biological specimens {26b}

Not applicable as no biological specimens were collected as part of this trial.

### Interventions

#### Explanation for the choice of comparators {6b}

The main comparator is microperimetry. It is justified by the fact that no study has shown that ILM peeling modifies postoperative VA but many studies have demonstrated anatomical damages. Visual outcome is not limited to VA. Microperimetry, a more refined test, can show microscotomas. These microscotomas do not impair VA but can cause permanent visual discomfort. Deltour et al. [23] showed in a retrospective study that active ILM peeling is associated with more microscotomas than spontaneous peeling. This preliminary result has to be verified by a prospective randomized clinical trial.

#### Interventions description {11a}

**Fig. 1:**
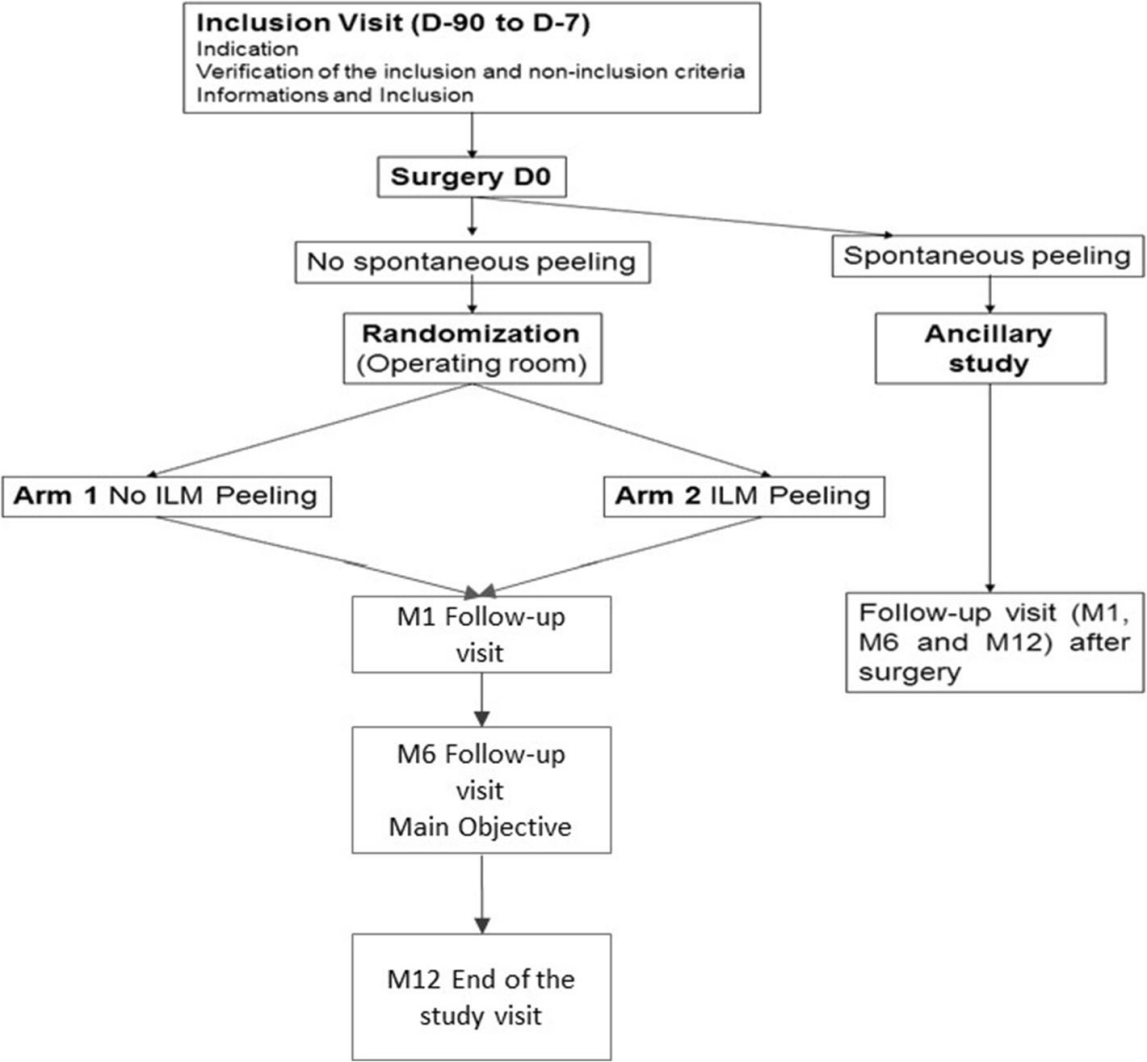
Study diagram

As shown in the study diagram (Fig. 1), the screening visit is conducted between day 90 and day 7 (D-90 to D-7) before surgery. It consists of an evaluation of the eye, VA and near vision, and SD-OCT analysis.

On the day of the surgery (D-0), for phakic eyes with cataract, phacoemulsification is performed. For all patients, central and peripheral vitrectomy (25 gauge) and dissection of the ERM are performed. Membraneblue-Dual® is used to stain ILM for an exposure time of 1 min. Intraoperative pictures are then taken to see the possible spontaneous ILM peeling: if ILM remains, the patient is randomized in the operating room in the “no peeling” group or in the “active peeling” group. In the “active peeling” group, the ILM peeling is performed on at least two papillary diameters around the fovea, which corresponds to a “circle” of four papillary diameters.

At the end of the surgery, the retinal periphery is checked. The operation is filmed and the anonymous videos are centralized in Nantes to compare the appearance of microscotomas and grip areas of the ERM and ILM seen by video and photo. For about 30% of patients [29], the ILM is spontaneously peeled off. These patients are not randomized but they can be included, if they want it, in the ancillary study. This ancillary study has the same follow-up visits (at 1, 6, and 12 months) with the same examinations as the principal study.

A total of three follow-up visits are planned over a time span of 12 months postoperatively (at 1, 6, and 12 months). At each visit, the following are performed: VA on the ETDRS scale; near vision (Parinaud scale); SD-OCT; microscopic examination of the eye; microperimetry; fundus photography and postoperative “Patient discomfort” questionnaire; biomicroscopic examination of the anterior segment; retinal photography; and an assessment of adverse events (AE). The schedule of the trial is shown in Table 2. To avoid any bias, the follow-up visits are made by masked ophthalmologists and orthoptists who do not know if ILM was actively peeled off.

**Table 2:** Study schedule

Actions	Inclusion visit D-90 and D-7	D0 (Surgery)	M1 (30days±7days starting D0)	M6 (6 months ± 15 days starting D0)	M12 (12 months ± 15 days starting D0) End of study
Patient information	X				
Informed consent	X				
History (medications taken…)	X				
Randomization		X			
Surgery		X			
ETDRS visual acuity score and near vision (Parinaud)	X		X	X	X
Evaluation of the appearance of the lens at the slit lamp	X				
Biomicroscopic examination of the anterior segment	X		X	X	X
Fundus	X		X	X	X
SD OCT	X		X	X	X
OCT/SLO: microperimetry	X		X	X	X
Retinal photography	X		X	X	X
“Patient discomfort” questionnaire	X		X	X	X
Adverse events		X	X	X	X
2 Photographs		X			
Video		X			

#### Criteria for discontinuing or modifying allocated interventions {11b}

The research could be discontinued as a result of the patient withdrawing their consent. The sponsor reserves the right to discontinue the study at any time for reasons that are well documented, especially in the case of unexpected AEs that compromise the safety of patients included in this study.

Finally, if the recruitment rate is too low or in cases of non-compliance with Good Clinical Practice, the study may also be stopped prematurely.

#### Strategies to improve adherence to interventions {11c}

This is a surgical procedure so patient adherence is not applicable.

#### Relevant concomitant care permitted or prohibited during the trial {11d}

Implementing “not-peeling” or “peeling” will not require alteration to usual care pathways (including use of any medication) and these will continue for both trial arms. In the postoperative phase, the standard treatment is a combination steroid and antibiotic eye drops (one drop three times per day) for the duration of 1 month.

#### Provisions for post-trial care {30}

At the end of the clinical research, the patient will be followed by their opthalmologist and will benefit from the usual care of their disease.

The sponsor takes out an insurance policy covering the financial consequences of its civil liability in compliance with the regulations.

#### Outcomes {12}

The main endpoint is the difference between the number of microscotomas (sensitivity < 10 dB) found before surgery and the number of microscotomas found at 6 months (number in the range of 0–29).

The secondary endpoints are:

- VA measured on the ETDRS scale and near vision (Parinaud) (at inclusion visit and 1, 6, and 12 months after surgery)
- Number and types of microscotomas by microperimetry (before and after surgery)
- Mean retinal sensitivity by microperimetry before and after surgery (at 1, 6, and 12 months)
- Patient visual discomfort questionnaire: symptoms and subjective improvement before and after surgery (at 1, 6, and 12 months)
- Incidence of changes of the retinal nerve fiber layer SD by OCT analysis (B scan and C scan) before and after surgery
- Sinusoid (IS/OS) line disruption by OCT analysis (B scan and C scan) before and after surgery
- Mean retinal thickness by OCT analysis (B scan) before and after surgery
- Retinal nerve fiber layer thickness by OCT analysis (B scan) before and after surgery
- Outer segment photoreceptor thickness by OCT analysis (B scan) before and after surgery
- Recurrence of ERM by OCT analysis (B scan and C Scan)
- Operative report and the video recording area of ERM and ILM gripping (study of the correlation with the microscotoma(s).

The endpoints of the ancillary studies are:

- The main criterion: the difference between the number of microscotomas (sensitivity < 10 dB) found before surgery and the number of microscotomas found at 6 months (number in the range of 0–29).
- VA measured on the ETDRS scale and near vision (Parinaud) (at inclusion visit and 1, 6, and 12 months after surgery)
- Number and types of microscotomas by microperimetry (before and after surgery)
- Mean retinal sensitivity by microperimetry before and after surgery (at 1, 6, and 12 months)
- Patient visual discomfort questionnaire: symptoms and subjective improvement before and after surgery (at 1, 6, and 12 months)
- Incidence of changes of the retinal nerve fiber layer SD by OCT analysis (B scan and C scan) before and after surgery
- Sinusoid (IS/OS) line disruption by OCT analysis (B scan and C scan) before and after surgery
- Mean retinal thickness by OCT analysis (B scan) before and after surgery
- Retinal nerve fiber layer thickness by OCT analysis (B scan) before and after surgery
- Outer segment photoreceptor thickness by OCT analysis (B scan) before and after surgery
- Recurrence of ERM by OCT analysis (B scan and C scan)

#### Participant timeline {13}

The treatment duration per patient corresponds to the surgery (1 day), the patient’s follow-up to 12 months and the recruitment period to 72 months.

#### Sample size {14}

A retrospective study conducted in the Department of Ophthalmology of the University Hospital of Nantes in 2013 [23] showed that of 11 patients who received active peeling, the number of microscotomas was 2.5 ± 3.1 preoperatively and 6.5 ± 7.3 at 1 month.

Based on the number of microscotomas found in the active peeling group in our retrospective study, with 80% power and a type I error of 5%, 100 patients are needed to highlight a halving of the number of microscotomas in the non-peeling group relative to the active peeling group (i.e. 6 ± 6 microscotomas average peeling in the active group compared to 3 ± 4.6 in the non-peeling group).

Usually, only very few patients do not attend their 6-month visit. However, the occurrence of EIGs (endophthalmitis, retinal detachment) may prevent the determination of the number of microscotomas at 6 months by microperimetry. Assuming that at most 5% of patients will be affected, 106 patients will be randomized, or 53 patients per group.

As all the patients with spontaneous peeling during surgery will not be randomized, > 106 have to be enrolled. The estimated number of spontaneous peeling was initially 30% [23], so the number of patients enrolled were thought to be 156. During the study, we noted that spontaneous peeling occurred in 50.2% of cases and so the number of patients to enroll in the study has been revised to 222 patients (amendment to the protocol in December 2017).

The ability of the six centers to recruit is estimated to be 120 patients per year (30 patients per center), given that the primitive ERM is a common disease.

#### Recruitment {15}

Recruitment is planned over a period of 72 months. Symptomatic idiopathic ERM is a common pathology found in each center at the rate of five cases per center per month, making these recruitment targets achievable.

### Assignment of interventions: allocation

#### Sequence generation {16a}

Randomization will be conducted openly and stratified by center. It will be performed according to a 1:1 ratio and balanced by blocks. The software used for the randomization is SAS version 9.4.

#### Concealment mechanisms {16b}

The random numbers will be generated by computer. Participants are randomized into blocks as the allocation progresses, a block being a subgroup of predetermined size within which there is a random allocation of patients.

#### Implementation {16c}

The randomization key is known only to the biostatistician and the data managers, to make it impossible for the investigator to assign a particular treatment.

During the surgery, if no spontaneous ILM peeling occurs, the patient is randomized at the block.

Patients are randomized into two groups

- Group 1: “no peeling” where the ILM peeling will not be made
- Group 2: “active peeling” where the ILM peeling is performed

### Assignment of interventions: blinding

#### Who will be blinded {17a}

The randomization is done during the surgery. To comply with the simple blind, the surgical team should not discuss the surgical procedure chosen in the operating block (so that the patient cannot hear).

Furthermore, to avoid any bias, the follow-up visits will be made by ophthalmologists and orthoptists who will not know what action has been carried out (masked team).

#### Procedure for unblinding if needed {17b}

To maintain the single blinding, the operative report will mention that the surgery was done following the protocol defined in the PEELING study. At the end of the study, patients will be informed of the results.

### Data collection and management

#### Plans for assessment and collections of outcomes: description of the parameters for evaluating efficacy {18a}

Here are detailed the parameters to assess effectiveness:

First, the microperimetry is a non-invasive test that allows a real-time, qualitative, and quantitative assessment of visual function. Microperimetry is introduced in routine clinical diagnostic procedure which, with extreme precision, defines the retinal attachment point and the threshold of differential sensitivity of the retina. Retinal sensitivity is better within 3° around the fixing point, with a mean foveal threshold of 20 dB, and a 0.275 dB mean decrement for each 10°. Scotomas are defined as absolute (if the patient does not perceive the maximum stimulation, the sensitivity is 0 dB) or relative (reduction of retinal sensitivity compared to normal values is < 10 dB). Assessing retinal function with this tool provides valuable clinical and pathophysiological information. These abnormalities could explain the hitherto non-assessable visual discomfort by conventional methods reported by some patients undergoing epiretinal membrane or macular hole surgery.

For all patients and to standardize the measurement, we use the “Ivana” protocol directed by the ophthalmology service of Lariboisière: a fixation target consisting of a red cross 2° in diameter, a white background monochromatic 4 asb, stimulus size Goldmann II with a projection time of 200 ms and a grid of 29 measurement points in the central 9° (centered on the fovea) with a 4–2-1 “double staircase” threshold strategy. Automatic “eye-tracking” will record eye movements throughout the exam. In addition, we will conduct topographies of the macular area in spectral OCT/SLO combined with microperimetry (OPKO/OTI, Miami, FL, USA).

Second, the scale of evaluation of the ETDRS VA will be performed by a masked orthoptist so as not to influence the patient’s response.

Third, a “Patient discomfort” questionnaire made by the ophthalmologist team will be given to the patient to evaluate their symptoms before and after the surgery.

Fourth, the SD-OCT will allow a precise anatomical pre- and postoperative analysis of the macula. The projected sequences include:

- RASTER (B scan): 1024 A scans; 25 sections (nine frames) spaced by 240 μm, covering an area of 20° × 20°
- FRONTAL acquisition (C scan): 512 A scans; 193 sections (16 frames) spaced by 30 μm, covering an area of 20° × 20°.

On these sections, we will study:

- The retinal thickness at 1000 μm central and the fovean crown at 3000 μm as well as the total macular volume
- The presence of intra-retinal and/or subretinal edema
- At the fovea level: analysis of internal trunk segments/outer segments of photoreceptors, integrity of the external limiting membrane, thickness of the layer of the outer section of the photoreceptors,
- In regard to microscotomas: thickness of the layer of ganglion cells at different times
- The rate of anatomical abnormalities that are detectable from the layer of optical fibers in frontal “DONFL” and “SANFL”
- The ERM rate of recurrence

Finally, the pre- and postoperative videos and photographs will be analyzed. The ERM peeling procedure with or without ILM is filmed. A photo or screen shot of the video just after staining with Membraneblue-Dual® and a photo or a video screen shot at the end of ILM peeling will be made (depending on the blocks, certain devices enable video only videos while others are able to take both videos and photos).

The objective of the first picture is to visualize the spontaneous ILM peeling and scope. The purpose of the second photo is to see the surface of active ILM peeling. The advantage of video is that the gripping zone of the ERM and ILM can be viewed.

Anonymous pre- and postoperative photos (according to the code defined in section 6.1.2.) will be sent to central Nantes and examined by an ophthalmologist from Nantes to see the gripping areas of the ERM and ILM.

#### Plan to promote participant retention and complete {18b}

In our current practice, we have noticed that very few patients refuse to attend the visit at 6 months that corresponds to the visit of the main objective (Fig. 1). A letter to remind them of the follow-up visit is sent 2 months before it. However, in case of no show or of serious adverse event (SAE; endophthalmitis, including retinal detachment), missing values for the primary endpoint will be dealt with multiple imputation*.* For the secondary endpoints, there will be no attribution of missing data.

#### Data management {19}

For each patient, a case report form (CRF) is created, which includes the data necessary to ensure compliance with the protocol and all data necessary for the statistical analysis and identify major protocol deviations. Data collection is done directly by the investigator or clinical research associate (CRA) in charge of the study, using an electronic CRF (eCRF) developed by the Promotion Department of the University Hospital of Nantes with ENNOV Clinical. The data are encoded to keep the identities of the patients confidential.

The collection of clinical data will be based on the establishment of a clinical database and the creation of input screens for image capture. The surgery videos are transferred anonymously on USB keys provided by the sponsor. A surgeon at the Department of Ophthalmology will note the gripping areas of the ERM in parallel with what the investigator has already noted.

#### Confidentiality {27}

Data collected during the study will be processed electronically in compliance with the requirements of the CNIL (compliance with the French Reference Methodology MR001). The CNIL is an independent French administrative regulatory body whose mission is to ensure that data privacy law is applied to the collection, storage, and use of personal data.

#### Plans for collection, laboratory evaluation, and storage of biological specimens for genetic or molecular analysis in this trial/future use {33}

Not applicable as no biological specimens were collected as part of this trial.

### Statistical methods

#### Statistical methods for primary and secondary outcomes {20a}

The variables measured at baseline are described for all patients in both group by numbers and percentages for each category for categorical variables and the minimum, maximum, average, standard deviation, and quartiles for the quantitative variables.

The primary endpoint is the difference between the number of microscotomas measured before surgery and the number of microscotomas measured at 6 months (a number in the range of 0–29). The mean difference is calculated in each of the two groups and compared using a mixed linear regression model to take into account the stratification of the randomization of the center (the center will be considered as a random effect) and adjustment for the preoperative number of microscotomas. Then, center-effect is analyzed with a fixed-effect model.

The following secondary endpoints are analyzed using a mixed linear regression model: comparison of the number of microscotomas in the two groups between preoperative visit and at 12 months; description and comparison of the type of microscotomas before surgery and at 1, 6, and 12 months; comparison of changes in mean visual acuity and in mean retinal sensitivity; study of the association between the presence of SANFL or DONFL with the number of microscotomas; comparison of the OCT B scan; and study of the associations between data in the OCT B scan and VA and between data in the OCT B scan and the presence of microscotomas.

The comparison between the two groups in the frequency of symptoms (blurred vision, metamorphopsia, relative scotoma, diplopia and micropsia), the comparison of the percentage of abnormalities detectable by OCT in front of the layer of optical fibers, and the comparison of the percentage of recurrence of the ERM at 12 months between the two groups are analyzed by Chi-square tests stratified on the center.

The correlation between VA and retinal sensitivity (the central point and the average of the five most central points) are analyzed according to the measurement of Pearson or Spearman’s correlation coefficients.

In addition, a description for each group of the number of microscotomas that are not located in a gripping area of the ERM and the ILM (at 1 month and 6 months) and a description of the outcome (disappearance/persistence) of microscotomas that existed before the surgery at 1 and 6 months is done.

The level of statistical significance is set at 0.05. Statistical analysis will be conducted in SAS software.

#### Interim analyses {21b}

No interim analysis will be performed and no early stopping rule for futility will be proposed.

#### Methods for additional analyses (e.g. subgroup analyses) {20b}

Analyses for the ancillary study. The patients with spontaneous peeling are not randomized and are compared to the “active peeling” group.

The main criterion is, like in the main study, the difference between the number of microscotomas (sensitivity < 10 dB) found before surgery and the number of microscotomas found at 6 months (number in the range of 0–29). The other endpoints are the same as the secondary endpoints of the main study, without the analysis of the video recording.

Mixed linear regressions models and stratified Chi-square test are used for comparisons.

#### Methods in analysis to handle protocol non-adherence and any statistical methods to handle missing data {20c}

As already stated very few patients generally refuse to attend the visit at 6 months. However, for the primary endpoint, in case of no show or of SAE (endophthalmitis, including retinal detachment), missing values will be dealt with multiple imputation.

Secondary endpoints: there will be no attribution of missing data for the secondary endpoints.

#### Plans to give access to the full protocol, participant level-data, and statistical code {31c}

According to French law, the results of the study will be published on the website of the regulatory authority. However, data sharing is prohibited by the General Data Protection Regulation European law.

### Oversight and monitoring

#### Composition of the coordinating center and trial steering committee {5d}

It has been possible to carry out the protocol and the trial thanks to an Executive Committee which includes a Scientific Committee and a Steering Committee. The Scientific Committee was created and the project manager of the clinical investigation center (CIC1413). The Steering Committee is composed of the members of the Scientific Committee with the addition of the data management team, the nurse study who coordinates assistance for patient inclusion in the other centers, and the monitoring CRA. The sponsor project manager coordinates this committee and drafts the “PEELING newsletter”, which provides, among other things, the latest news on patient inclusion, amendments to the protocol, etc.

#### Composition of the data monitoring, its role, and reporting structure {21a}

This surgery is done in routine practice; therefore, a Data and Safety Monitoring Committee was irrelevant.

#### Adverse event reporting and harms {22}

Expected AEs as a result of epiretinal membrane and cataract surgery (according to the Société Française d’Ophtalmologie, www.sfo.asso.fr) are:

- endophthalmitis (eye infection) (1–3 in 1000)
- changes in the macula, tear(s) of the retina, retinal detachment (3%) that may occur after surgery and require additional treatment by reoperation and/or laser
- clouding of the cornea
- central retinal edema
- retinal burn from the illumination of the surgical microscope
- inadequately sealed scar
- partial collapse of the upper eyelid
- subconjunctival hemorrhage or of eyelid
- perception of floaters
- increased sensitivity to light
- inflammation of the eye
- increased intra-ocular pressure

All SAEs, whether expected or unexpected, require the completion of a SAE report. The investigator should verify that all the information noted in this report is precise and clear (no abbreviations, etc.).

All the surgery-related AEs occurring inside the operating room must be reported in the eCRF and, if they meet a seriousness criterion, transmitted to the sponsor.

Because they could be a risk of adverse drug reaction, any inadequacy or malfunction of a medical device or surgical equipment has to be notified to the sponsor as well as any misuse or error.

SAEs should be reported immediately (within 24 h of the investigator becoming aware of the event) to the sponsor by fax (Research Department, CHU de Nantes – Fax number: + 33 2 53 48 28 36).

On receipt of an unexpected SAE report, the sponsor should report it to the regulatory authorities. Once a year, the sponsor draws up an annual safety report.

In the event of SAEs involving discontinuing from the study or SAEs ongoing at the end of the study, the patient should be subject to follow-up until the SAE is resolved.

#### Frequency and plans for auditing trial conduct {23}

An inspection or audit may take place as part of this study, performed by the sponsor and/or by the regulatory authorities. Inspectors will check the documents, logistics, records, and any other resources that the authorities consider to be associated with the clinical trial and that may be located at the trial site itself.

#### Plans for communicating important protocol amendments to relevant parties (e.g. trial participants, ethical committees) {25}

The amended protocol will be a dated, updated version. If necessary, the information form and consent form should be amended. The sponsor project manager will notify the centers and a copy of the revised protocol will be sent to all the principal investigators to add to the Investigator Site File. Currently, the updated protocol is at version 7 on 6 February 2020. All the submissions/declarations were made by the Sponsor Department at CHU Nantes to the French regulatory authority (ANSM) and the ethic committee (Comité de Protection des Personnes OUEST IV – Nantes). The CRA of the Sponsor Department will report any deviations of the protocol. They will be fully documented using a breach report form.

#### Disseminations plans {31a}

The trial results will be published in international ophthalmological, medical, and scientific journals and presented at national and international conferences.

## Discussion

The peeling of the ILM is frequently practiced by surgeons during ERM surgery to reduce recurrences of ERM. It does not impair nor improve postoperative VA, but it increases the surgical risks and causes anatomical damages. This procedure may cause definitive visual discomfort which patients often complain about.

PEELING is designed to explore this aspect by comparing the difference in number of microscotomas whether the patients had their ILM peeled or not. The intention is to assess the benefit/risk ratio of ILM peeling. Recurrence of ERM, VA, patient’s visual discomfort, and various anatomical endpoints of the retina are also evaluated.

After receiving appropriate approval, the study included its first patient in September 2014. The study is conducted for 72 months; inclusions will stop as of September 2020. This recruitment period had to be extended as a result of more spontaneous peeling of the ILM during surgery compared to the proportion that was statistically planned.

This study is a single-blind trial considering the impossibility of blinding the surgeon during the practice of the ILM peeling. However, to limit any more bias, the follow-up visits are conducted by blinded ophthalmologists and orthoptists who do not know what action has been carried out.

Two features are particularly original in this study: randomization in the operating room and microperimetry as the primary endpoint.

On the one hand, randomization in the operating room fits with the everyday pratice when the surgeon has removed ERM and is assessing remaining ILM. It allows the prospective measurement of the “spontaneous peeling” rate and it generates a large group of > 100 patients included in the ancillary study. The comparisons of this group with the two groups—“active peeling” and “no peeling”—will elucidate the clinical significance of this phenomenon that has been largely neglected.

On the other hand, choosing microperimetry as the primary endpoint is unusual but crucial. It is justified by the fact that no study has yet shown that ILM peeling modifies postoperative VA but many studies have demonstrated anatomical damages. Visual outcome can not be limited to VA and more refined tests are needed to evaluate if these anatomical damages and associated with visual impairment. Deltour et al. [23] showed in a retrospective study that active ILM peeling is associated with more microscotomas than spontaneous peeling. This preliminary result has to be verified by a prospective randomized clinical trial.

With this study, the expected individual benefit is an improved visual comfort of patients operated for idiopathic ERM. If this study shows that active ILM peeling is significantly associated with more microscotomas and patient discomfort, it may contraindicate the ILM peeling during primitive idiopathic ERM surgery.

### Trial status

This trial is still ongoing; patient inclusion is not yet complete.

The updated protocol is at version 7 on 6 February 2020.

The first patient was included on 9 September 2014.

Recruitment by the investigating centers is planned to continue until September 2020.

## Supplementary information

**Additional file 1.** Informed consent form. The informed consent form given to each patient (French version).

## Data Availability

Data sharing is not applicable to this paper as no datasets were generated or analyzed during the current study. The investigators will share the entirety of the final trial dataset. Data collected during the test may be processed electronically, in accordance with the requirements of the CNIL (compliance with reference methodology MR001).

## Abbreviations

AE: Adverse event
AMD: Age-related macular degeneration
ANSM: Agence Nationale de Sécurité du Médicament et des produits de santé – French regulatory authorities
CNIL: Commission Nationale de l’Informatique et des Libertés – French National Commission on Informatics and Liberty
CRA: Clinical Research Associate
CRF: Case report form
DONFL: Dissociation of optic nerve fiber layer
ERM: Epiretinal membrane
ILM: Internal limiting membrane
OCT: Optical coherence tomography
SD-OCT: Spectral domain optical coherence tomography
SAE: Serious adverse event
SANFL: Swelling of the arcuate nerve fiber layer
SLO: Scanning laser ophthalmoscope
