# Supplementary material for: Does internal limiting membrane peeling during epiretinal membrane surgery induce microscotomas on microperimetry? Study protocol for PEELING, a randomized controlled clinical trial
Source: Trials. 2020 Jun 8;21:500. doi: 10.1186/s13063-020-04433-9 (PMC7278143; doi:10.1186/s13063-020-04433-9)
Supplement: Supplementary file 1 — Additional file 1. Informed consent form. The informed consent form given to each patient (French version). [file 13063_2020_4433_MOESM1_ESM.doc]

Note d’information patient

**Note d’information pour la participation à la recherche**

**«**Etude prospective randomisée multicentrique contrôlée en simple insu, évaluant l’intérêt du pelage de la membrane limitante interne lors de la chirurgie des membranes épimaculaires **»**

**Titre abrégé : « Etude randomisée évaluant l’intérêt du pelage de la membrane limitante interne lors de la chirurgie des membranes épimaculaires »**

***Promoteur : CHU de Nantes***

***N° EudracT/ Enregistrement :* 2014-A00202-45**

***Promoteur : CHU de Nantes***

***N° EudracT/ Enregistrement :* 2014-A00202-45**

Madame, Monsieur,

Dans le cadre de la prise en charge de votre maladie, *la membrane épi rétinienne*, (encore appelé membrane prététinienne ou membrane épimaculaire) nous vous proposons de participer à une recherche biomédicale nommée « **Etude randomisée évaluant l’intérêt du pelage de la membrane limitante interne lors de la chirurgie des membranes épimaculaires**». Cette recherche a pour but de comparer deux procédures chirurgicales traitant les membranes épi rétiniennes.

Le CHU de Nantes est le promoteur de cette recherche, c’est à dire qu’il en est responsable et qu’il l’organise.

**COMMENT VOUS DECIDER ?**

Votre médecin-investigateur vous a donné des explications, Toutes ces explications en lien avec cette étude, sont retranscrites dans ce document intitulé « note d’information ». Nous vous invitons à le lire attentivement avant de vous décider. Prenez votre temps avant de décider de votre participation à cet essai clinique.

Si vous décidez de participer à cette recherche, on vous demandera de signer une attestation de consentement. Cette attestation sera aussi signée par votre médecin. Cette signature confirmera que vous êtes d’accord pour participer à la recherche. Votre signature est indispensable, ainsi que celle du médecin. Même après avoir signé pour donner votre accord de participation, vous garderez le droit d’interrompre à tout moment votre participation sans avoir à vous justifier.

**QUE SAIT-ON DEJA SUR LES MEMBRANES EPI RETINIENNES ?**

Vous êtes atteint(e) d'une membrane épi rétinienne maculaire responsable de vos troubles visuels (flou visuel, baisse d’acuité visuelle ± déformation des lignes). Votre ophtalmologiste vous propose l'opération, car la chirurgie constitue le seul moyen d'améliorer votre vision.

**La membrane épi rétinienne maculaire :** elle correspond à une très mince pellicule de tissu qui recouvre et plisse le centre de la rétine appelé la macula. Il s'agit d'une pathologie habituellement liée au vieillissement de l'oeil. D'autres causes, plus rares, sont possibles.

**Pourquoi opérer la membrane épi rétinienne?** Parce que cette membrane peut entraîner une aggravation progressive des déformations visuelles qui deviennent de plus en plus gênantes, ou finir par entraîner une baisse progressive de l'acuité visuelle centrale. Cependant cette affection n'entraîne jamais de cécité complète.

**La chirurgie des membranes épi rétiniennes :** l’intervention a lieu le plus souvent sous anesthésie locorégionale (injections de produit anesthésiant autour de l’œil) moins souvent sous anesthésie générale. Le choix résulte de l’avis de votre ophtalmologiste et de celui du médecin anesthésiste. Il prend en compte, si possible, votre souhait.

Le mode d'hospitalisation adapté à votre cas vous sera proposé par votre ophtalmologiste en accord avec l'anesthésiste.

L'intervention est réalisée alors que vous êtes installé sur le dos, en milieu chirurgical stérile et sous microscope.

Elle consiste, par l’intermédiaire de très petits instruments à retirer dans un premier temps le vitré (gel transparent à l’intérieur de l’œil) puis dans un second temps, la membrane épi rétinienne à l'aide d’une micro-pince.

L’intervention dure environ 1/2 heure.

Si votre médecin le juge nécessaire une opération de la cataracte (cristallin ayant perdu sa transparence) sera faite en même temps que la chirurgie des membranes épimaculaires.

Cette chirurgie permet de façon générale d’améliorer l’acuité visuelle dans ¾ des cas sans pour autant toujours permettre de normaliser votre acuité visuelle. Elle permet aussi de diminuer dans la grande majorité des cas la déformation des lignes lorsqu’elle est présente. Le bénéfice visuel se produit de façon progressive sur une période de plusieurs mois.

Bien qu'elle soit, dans la grande majorité des cas, suivie de bons résultats, l'opération des membranes épi rétiniennes n'échappe pas à la règle générale selon laquelle il n'existe pas de chirurgie sans risque.

**QUEL EST LE BUT DE CETTE RECHERCHE ? QUEL EST SON DEROULEMENT GENERAL ?**

La chirurgie des membranes épi rétiniennes est réalisée depuis de nombreuses années. Pour réduire le risque de récidive de ces membranes, les chirurgiens pratiquent de plus en plus souvent, l’ablation complémentaire d’une fine membrane qui se situe sous la membrane épi rétinienne et la partie la plus superficielle de la rétine nommée la « membrane limitante interne de la rétine ».

Nous voulons vous proposer de participer à une étude clinique qui nous permettra d’évaluer le bénéfice mais aussi les inconvénients de ce pelage de membrane limitante interne.

Le bénéfice serait une réduction du risque de récidive qui demeure de toute façon faible (d’environ 5%).

L’inconvénient du pelage de cette membrane limitante pourrait être de générer parfois un inconfort visuel.

L’objectif de cette étude est d’évaluer le rapport entre le bénéfice et l’inconvénient de la réalisation d’un pelage de membrane limitante interne pour tenter d’améliorer encore un peu plus le traitement de ces membranes épi rétiniennes

Cette étude nationale s’effectue sur sept centres : les services d’ophtalmologie du CHU de Nantes (Pr Michel WEBER), de L’Hôpital Lariboisière et de l’hôpital Cochin (Pr Ramin TADAYONI) et du CHU de Dijon (Pr Catherine CREUZOT GARCHER) et de la fondation Rothschild (Dr Yannick LE MER), de la Polyclinique de l’Atlantique de Saint-Herblain (Dr Julien Perol , Dr Alexandre Bourhis, Dr François Lignereux) et de la Clinique Sourdille de Nantes Dr Frank Becquet). Elle concerne 222 patients nécessitant comme vous une chirurgie de membrane épi rétinienne avec 106 patients répartis par tirage au sort en deux groupes :

- Dans un des groupes le chirurgien réalisera l’ablation de la fine membrane sous jacente nommée « membrane limitante interne de la rétine ».

- Dans le second groupe ce geste ne sera pas réalisé. Vous ne serez pas informé de la technique chirurgicale qui a été réalisée lors de votre intervention.

Pour certains patients, il arrive que lors de la chirurgie, le geste élimine de manière spontanée la membrane limitante interne On appelle ce processus un pelage spontané de la membrane. Ces patients ne seront pas répartis par tirage au sort car aucun acte chirurgical complémentaire n’est nécessaire. On vous proposera le même suivi dans ce cas (dans le cadre d’une étude dite ancillaire).

Tous les patients auront les mêmes visites et les mêmes examens.

**QU’ARRIVERA T-IL PENDANT LA RECHERCHE ? QU’AUREZ-VOUS A FAIRE ?**

**Votre participation éventuelle à cette recherche durera 1 an.**Votre accord pour participer à cette recherche demandera de votre part un engagement pendant toute la durée de la recherche à vous rendre à toutes les visites prévues dans le protocole :

- **La visite de sélection/inclusion :** pourra être scindée en deux visites en cas d’obligations du médecin et/ou de vous-même. Cette visite permettra d’établir précisément le diagnostic de membrane épi rétinienne et juger de l’indication chirurgicale. L’interrogatoire et l’examen clinique ophtalmologique permettront d’évaluer si vous répondez aux critères d’inclusion et de non inclusion. Si vous répondez aux critères, on vous présentera cette recherche clinique. Cette notice d’information vous sera alors remise, puis après un délai de réflexion, si vous souhaitez participer, il vous sera demandé de signer le formulaire de consentement de participation à cette étude.

Un bilan clinique ophtalmologique complet sera pratiqué. Vous devrez remplir le questionnaire « Inconfort du patient ». Un examen par micropérimétrie sera également effectué. Il nous permettra d’évaluer objectivement votre qualité de vision.

- **Le jour même de la chirurgie**, l’investigateur sera informé, après tirage au sort, si la réalisation ou non du pelage de la membrane limitante interne devra être réalisée. Vous ne serez pas si le pelage ou non de la membrane limitante interne a été réalisé.Dans les cas où le pelage de cette membrane limitante interne se fait spontanément, vous n’aurez aucun acte chirurgical supplémentaire.
- **En post opératoire 3 consultations de contrôle sont nécessaires : à 1 mois, 6 mois puis à 1 an de la chirurgie :** vous devrez remplir le questionnaire « Inconfort du patient » et un examen ophtalmologique complet ainsi qu’une micropérimétrie seront faits.

**QUELS SONT LES RISQUES ?**

**Quels sont les risques que vous courez si vous participez à cette étude ?**

**Cette recherche ne présente aucun risque de plus que la prise en charge habituelle** qui vous serait proposée, si vous ne participiez pas à la recherche. Vous devrez de toute façon être prise en charge de manière chirurgicale. Quelle que soit la technique opératoire, la membrane épi maculaire sera enlevée. Le reste de la prise en charge restera identique à celle habituellement pratiquée.

Les risques liés à la chirurgie de membrane épi maculaire vous sont détaillés ci-dessous et dans la fiche d’information N°14 « Virectomie pour menbrane prémaculaire »). Ils sont équivalents quel que soit la procédure chirurgicale décidée le jour de l’intervention.

Les complications sévères de l'opération de vitrectomie pour membrane épi rétinienne sont très rares. Elles peuvent nécessiter une réintervention et aboutir, dans les cas les plus extrêmes, à la perte de toute ou partie de la vision de l'œil opéré, voire à la perte de l'œil lui-même. Il s'agit de :

- déchirure(s) de la rétine, décollement de la rétine pouvant survenir après l'intervention et nécessiter un traitement complémentaire par réintervention chirurgicale et/ou laser,

- infection oculaire,

- altération de la macula.

D'autres complications sont moins sévères et le plus souvent transitoires comme :

- chute partielle de la paupière supérieure,

- hémorragie ou hématome de la partie externe de l'œil (blanc de l’œil) ou de la paupière,

- perception de mouches volantes.

Dans les suites post-opératoires, vous devez consulter en urgence votre chirurgien ou à défaut votre ophtalmologiste en cas de signes d’alerte, pouvant faire évoquer une complication, tels que :

- Baisse d’acuité visuelle par rapport au lendemain de l’intervention,

- Douleurs oculaires,

- Apparition ou augmentation d’une rougeur du globe oculaire.

En outre et comme toute chirurgie des complications, du fait de l’anesthésie, peuvent survenir.

Vous trouverez des informations complémentaires notamment sur les résultats et les risques de l’opération « Vitrectomie pour membrane prémaculaire » sur la fiche d’information de cette opération que votre médecin vous a remis pour la prise en charge de votre chirugie (cf.Vitrectomie pour membrane prémaculaire SFO Fiche d’information n°14 version novembre 2009)

En cas de problème, et à tout moment dans la recherche, vous devrez contacter votre médecin-investigateur.

Votre médecin traitant sera, avec votre accord, informé de votre participation à cette étude.

**QUELS SONT LES BENEFICES QUE VOUS POUVEZ ESPERER ?**

- **Bénéfices personnel thérapeutique attendu**

Vous n’aurez personnellement aucun bénéfice thérapeutique direct en participant à l’étude. La durée opératoire et la durée d’hospitalisation ne seront pas modifiées par la technique chirurgicale utilisée. Cependant il semble que le confort visuel post opératoire est meilleur en cas de non pelage de la membrane limitante interne.

- **Bénéfice personnel non thérapeutique attendu**

Vous bénéficiez d’un suivi post opératoire comprenant un examen non réalisé en pratique courante et non douloureux : la micropérimétrie. Il permettra d’évaluer précisément votre confort visuel.

- **Bénéfice collectif attendu :**

La participation volontaire à une recherche biomédicale est utile à tous. Cette étude scientifique pourrait ainsi influer sur les futures pratiques chirurgicales et améliorer le résultat fonctionnel post opératoire des patients.

**QUELLES SONT LES ALTERNATIVES ?**

Si vous ne souhaitez pas participer à cette étude, une prise en charge chirurgicale vous sera proposée pour prendre en charge votre membrane épi rétinienne. La durée d’hospitalisation et le suivi post opératoire sera identique à celui prévu dans cette étude. Vous n’aurez cependant pas à effectuer des examens par microperimétrie lors de vos visites de contrôle.

**QUE SE PASSERA-T-IL A LA FIN DE LA RECHERCHE, SI LA RECHERCHE S'ARRETE OU SI VOUS DECIDEZ D’INTERROMPRE VOTRE PARTICIPATION ?**

La recherche peut être interrompue à tout moment:

- par les autorités de santé,
- du fait du promoteur, le CHU de Nantes : si un élément nouveau survient, le médecin-investigateur en sera informé et il vous transmettra alors les éléments susceptibles de modifier votre participation,
- par vous-même : si vous décidez de participer à cette recherche, il s’agira d’un acte volontaire. Vous pourrez à tout moment décider d’arrêter votre participation, sans pénalité ni préjudice. Dans ce cas, vous devez informer le médecin-investigateur de votre décision.

Quelle que soit la raison de votre interruption, le médecin-investigateur vous informera alors des mesures à suivre. Dans tous les cas, la qualité de votre prise en charge ne sera pas diminuée.

**AUREZ-VOUS DES FRAIS SUPPLEMENTAIRES ?**

Votre participation à cette recherche n'engendrera pour vous aucun frais supplémentaire par rapport à ceux que vous auriez pour la prise en charge habituelle de cette maladie.

Si besoin, une aide à vos frais de déplacement pourra être envisagé, dans la limite de 100€ / visite pour la visite d’inclusion (si cette dernière est scindée en deux), pour la visite à M6 et M12. La consultation initiale et la consultation un mois après la chirurgie ne seront pas concernés par cette aide car elles font parties du suivi habituel.

**QUELS SONT VOS DROITS PENDANT LA RECHERCHE ?**

Le personnel impliqué dans la recherche est soumis au **secret professionnel**, tout comme votre médecin traitant.

**Accès aux données vous concernant**- Traitement des données-CONFIDENTIALITE

Dans le cadre de cette recherche, un traitement informatique de vos données personnelles va être mis en oeuvre : cela permettra d’analyser les résultats de la recherche et de remplir l’objectif de la recherche.

Pour cela, les données médicales vous concernant (et les données relatives à vos habitudes de vie), seront transmises au Promoteur de la recherche (CHU de Nantes) ou aux personnes ou sociétés agissant pour son compte. Ces données seront identifiées par un numéro de code et vos initiales.

Ces données pourront également, dans des conditions assurant leur confidentialité, être transmises aux autorités sanitaires habilitées.

Si vous décidez de retirer votre consentement pour participer à la recherche, les données obtenues avant que celui-ci n'ait été retiré seront utilisées. Les données recueillies après le retrait de votre consentement ne seront pas utilisées pour cette recherche et resteront destinées à l’usage strict du soin.

Elles seront susceptibles d’être exploitées dans le cadre de publications ou de communications; dans ce cas, votre anonymat sera préservé.

Conformément aux dispositions de la loi relative à l’informatique aux fichiers et aux libertés (loi modifiée du 6 janvier 1978), de la loi n° 2018-493 du 20 juin 2018 relative à la protection des données personnelles et du Règlement (UE) 2016/679 du Parlement européen et du Conseil du 27 avril 2016 relatif à la protection des personnes physiques à l'égard du traitement des données à caractère personnel et à la libre circulation de ces données (RGPD), vous disposez d’un droit d’accès, de rectification, et de limitation du traitement de vos données personnelles. Vous pouvez également porter une réclamation auprès d'une autorité de contrôle (CNIL pour la France : <https://www.cnil.fr/fr/webform/adresser-une-plainte/>).

Ces droits s’exercent auprès du médecin-investigateur qui vous suit dans le cadre de la recherche et qui connaît votre identité.

Vous pouvez également accéder directement ou par l’intermédiaire d’un médecin de votre choix à l’ensemble de vos données médicales en application des dispositions de l’article L 1111-7 du Code de la Santé Publique.

Vos données seront conservées tout au long de la recherche. Après la fin de la recherche, vos données seront archivées pour une durée conforme aux dispositions réglementaires, puis détruites.

Pour en savoir plus ou exercer vos droits concernant vos données, voir vos contacts en annexe 1.

**QUELLES SONT VOS OBLIGATIONS PENDANT LA RECHERCHE ?**

**Vos obligations :**

Vous devez informer votre médecin investigateur de tous les médicaments que vous prenez.

Vous devez aussi l’informer immédiatement de tout effet indésirable éventuellement rencontré au cours de votre participation à la recherche.

Vous devez vous rendre aux visites prévues.

**Protection sociale :**

Pour pouvoir participer à cette recherche vous devez être affilié(e) ou bénéficier d’un régime de sécurité sociale *(CMU acceptée)*.

**Modalités de participation à une autre recherche :**

Pendant toute la durée de l’étude, la participation à une autre recherche clinique n’est pas possible.

**LE CADRE REGLEMENTAIRE**

**Cette recherche est conforme :**

- Aux articles L. 1121-1 à L. 1126-12 du code de la santé publique relatifs aux recherches *impliquant la personne humaine*

- A la loi « Informatique et Libertés » du 6 janvier 1978 modifiée et *la loi n° 2018-493 du 20 juin 2018 relative à la protection des données personnelles*

- au *Règlement (UE) 2016/679 du Parlement européen et du Conseil du 27 avril 2016 relatif à la protection des personnes physiques à l'égard du traitement des données à caractère personnel et à la libre circulation de ces données (RGPD)*

Vous pouvez retrouver tous ces textes sur le site [http://www.legifrance.gouv.fr](http://www.legifrance.gouv.fr/)

**Conformément aux dispositions réglementaires :**

Le CHU de Nantes organise cette recherche en tant que « promoteur ». Il a souscrit un contrat d’assurance garantissant sa responsabilité civile et celle de tout intervenant auprès de la compagnie SHAM (contrat n°127 609).

Cette recherche a reçu l’avis favorable du Comité de Protection Ouest IV le 01/04/2014

La recherche a aussi reçu l’autorisation de l’ANSM (Agence Nationale de Sécurité du Médicament et des Produits de Santé), le 21/02/2014

**EN RESUME**

Votre participation à cette recherche est libre. Vous pouvez refuser de participer à cette recherche.

De plus, vous pouvez à tout moment vous retirer de cette recherche, sans préjudice.

Si vous décidez de refuser de participer à la recherche ou si vous décidez d’arrêter votre participation pendant la recherche :

- cela n’aura aucune conséquence sur la qualité des soins qui vous seront donnés
- vous devez simplement en informer votre médecin-investigateur.

Lorsque vous aurez lu cette note d’information et obtenu les réponses aux questions que vous vous posez en interrogeant le médecin-investigateur. Il vous sera proposé, si vous en êtes d’accord, de donner votre consentement écrit en signant le formulaire préparé à cet effet.

Si vous acceptez de participer à la recherche, il faudra rapporter votre formulaire de consentement signé, au plus tard, lors de la prochaine visite.

Vous pouvez prendre votre temps avant de nous donner votre réponse.

Au cours de ce délai de réflexion, vous pouvez bien entendu continuer par téléphone à poser toutes les questions que vous souhaitez à votre médecin, le Pr/Dr ………………au ………………

Nous vous prions d’agréer, Madame, Mademoiselle, Monsieur, l’expression de nos sentiments les plus respectueux.

Pr Weber Michel (service d’ophtalmologie du CHU de Nantes) médecin coordonnateur et investigateur principal, et toute l’équipe médicale en charge de cette recherche

*Vous devez conserver un exemplaire de ce document.*

**ANNEXE**

# ANNEXE 1 : LES CONTACTS IMPORTANTS :

**Pour toute question concernant l’étude, retrait de consentement, ou pour exercer vos droits concernant vos données (accès rectification, etc… ) :**

**L’investigateur coordonnateur de la recherche :**

Prof. Michel Weber

Department: Ophthalmology

Address: CHU DE Nantes

Place Alexis Ricordeau 44093 Nantes cedex 1.

Tel: 02.40.08.34.06

Email michel.weber@chu-nantes.fr

# Pour toute question générale sur la protection de vos données :

**Le promoteur de la recherche, responsable du traitement :**

CHU de Nantes, direction de la recherche

5 allée de l’Ile Gloriette, 44093 NANTES Cedex 1

**Le Délégué à la protection des Données (DPO) :**

*vosdonneespersonnelles@chu-nantes.fr*

CONSENTEMENT ECLAIRE DE PARTICIPATION A L’ETUDE :

**« Etude prospective randomisée multicentrique contrôlée en simple insu, évaluant l’intérêt du pelage de la membrane limitante interne lors de la chirurgie des membranes épimaculaires »**

**Protocole référencé N°RC14_0026**

Je soussigné (e), (Ajouter vos nom & prénom en lettres majuscules) ……………………………………………………….……………………………………………………………,

Né(e) le *|__|__| |__|__| |__|__|__|__|*

certifie être affilié(e) à un régime de sécurité sociale et **accepte librement et volontairement de participer à la recherche** **référencée ci-dessus,** coordonnée par le Professeur Weber et organisée par le CHU de Nantes, promoteur de la recherche.

Je déclare avoir pris connaissance de la notice d’information et avoir bien compris les informations qui m’ont été précisées par le Docteur …………….……………………concernant l’étude ci-dessus mentionnée et à laquelle j’accepte de participer.

J'ai lu le formulaire d'information qui m'a été remis (pages 1 à 9) et bien compris les informations suivantes : l'objectif de la recherche, sa méthodologie et sa durée, les bénéfices attendus, les contraintes et les risques prévisibles de cette recherche. J'ai eu l'opportunité de poser toutes les questions concernant cet essai et reçu des réponses satisfaisantes.

Il m’a été précisé que je suis libre d’accepter ou de refuser de participer à cette recherche, sans que cela ne modifie ma prise en charge médicale. Je pourrai, à tout moment, si je le désire, interrompre ma participation en le signalant au médecin chargé de cette étude, sans encourir la moindre responsabilité et sans aucun préjudice pour la qualité des soins qui me seront prodigués.

Mon consentement ne décharge pas les responsables de la recherche de leurs responsabilités. Je conserve tous mes droits garantis par la loi. Pour tout complément d’information ou pour signaler la survenue d’un événement indésirable, je peux contacter le médecin chargé de l’étude.

J’accepte que les données collectées au cours de cette étude puissent faire l’objet d’un traitement informatisé par le Pr Weber ou les personnes agissant pour son compte. Les données qui me concernent resteront strictement confidentielles. Je n’autorise leur consultation que par des personnes qui collaborent à la recherche ou qui sont mandatées par le Pr Weber et par un représentant des autorités de santé; toutes ces personnes étant soumises au secret professionnel.

J’atteste avoir été informé de tous mes droits concernant mes données personnelles selon les modalités décrites dans la note d'information en vigueur, qui m'a été transmise pour ce protocole.

J’ai pu poser toutes les questions que je voulais et j’ai reçu des réponses adaptées. J’ai disposé d’un temps de réflexion suffisant entre l’information et le consentement.

Fait à …………….., le …../…./…..  *Signature du patient*

Je déclare avoir remis le formulaire d'information au patient et accuse réception du consentement éclairé.

Fait à …………….. le ..…./…../…… *Nom et Signature de l’investigateur*

**Ce document est à réaliser en 2 exemplaires originaux : le premier doit être conservé par l’investigateur et le deuxième est remis à la personne donnant son consentement. En cas de duplicata, l’original est conservé par l’investigateur et une copie est remise à la personne ayant donné son consentement.**
